# Clinical prevalence of Lewy body dementia

**DOI:** 10.1186/s13195-018-0350-6

**Published:** 2018-02-15

**Authors:** Joseph P. M. Kane, Ajenthan Surendranathan, Allison Bentley, Sally A. H. Barker, John-Paul Taylor, Alan J. Thomas, Louise M. Allan, Richard J. McNally, Peter W. James, Ian G. McKeith, David J. Burn, John T. O’Brien

**Affiliations:** 1 Institute of Neuroscience, Biomedical Research Building, Campus for Ageing and Vitality, Newcastle University, Newcastle upon Tyne, NE4 5PL UK; 2 Department of Psychiatry, University of Cambridge School of Clinical Medicine, Box 189, Level E4 Cambridge Biomedical Campus, Cambridge, CB2 0SP UK; 3 Institute of Health and Society, Sir James Spence Institute, Royal Victoria Infirmary, Newcastle University, Queen Victoria Road, Newcastle upon Tyne, NE1 4LP UK

**Keywords:** Dementia with Lewy bodies, Dementia in Parkinson’s disease, Epidemiology, Prevalence

## Abstract

**Background:**

The prevalence of dementia with Lewy bodies (DLB) and dementia in Parkinson’s disease (PDD) in routine clinical practice is unclear. Prevalence rates observed in clinical and population-based cohorts and neuropathological studies vary greatly. Small sample sizes and methodological factors in these studies limit generalisability to clinical practice.

**Methods:**

We investigated prevalence in a case series across nine secondary care services over an 18-month period, to determine how commonly DLB and PDD cases are diagnosed and reviewed within two regions of the UK.

**Results:**

Patients with DLB comprised 4.6% (95% CI 4.0–5.2%) of all dementia cases. DLB was represented in a significantly higher proportion of dementia cases in services in the North East (5.6%) than those in East Anglia (3.3%; χ^2^ = 13.6, *p* < 0.01). DLB prevalence in individual services ranged from 2.4 to 5.9%. PDD comprised 9.7% (95% CI 8.3–11.1%) of Parkinson’s disease cases. No significant variation in PDD prevalence was observed between regions or between services.

**Conclusions:**

We found that the frequency of clinical diagnosis of DLB varied between geographical regions in the UK, and that the prevalence of both DLB and PDD was much lower than would be expected in this case series, suggesting considerable under-diagnosis of both disorders. The significant variation in DLB diagnostic rates between these two regions may reflect true differences in disease prevalence, but more likely differences in diagnostic practice. The systematic introduction of more standardised diagnostic practice could improve the rates of diagnosis of both conditions.

## Background

Dementia with Lewy bodies (DLB) is a common cause of dementia in older people, characterised by a tetrad of visual hallucinations, fluctuations in cognition, spontaneous parkinsonism, and REM sleep behaviour disorder. Parkinson’s disease dementia (PDD) describes dementia arising in the context of established idiopathic Parkinson’s disease (PD), and shares both neurobiological and clinical characteristics with DLB. Together, DLB and PDD comprise Lewy body dementia (LBD), conceptualised as a spectrum disorder associated with cortical and subcortical Lewy body pathology, with variations in the temporal onset of motor and cognitive symptoms [1–3].

Validated diagnostic criteria [2] and clinical biomarkers exist for DLB [4, 5]. However, despite the important implications of diagnosis for treatment, mortality [6], and carer well-being [7], previous studies have suggested that only one in three cases is correctly identified in routine clinical care [8, 9] and a considerable lack of consensus surrounds the actual prevalence of DLB.

A recent meta-analysis of epidemiological studies reported that DLB represented 7.5% of all dementia cases in clinical populations [10]. These populations refer to research cohorts in which consecutive referrals to a service or healthcare organisation were screened for DLB on the basis of clinical symptoms and investigations. The same meta-analysis found that DLB comprised 4.2% of community-based dementia populations. However, studies contributing to this meta-analysis observed prevalence rates ranging from 0 to 26% in individual cohorts [11, 12].

Variation between individual studies’ prevalence rates could represent true differences in DLB prevalence among different regions or countries. However, the wide range of methodological and sampling practices adopted in these studies is an alternative cause for the reported rates.

There is a greater consensus regarding the prevalence of PDD. A systematic review in 2005 found the point prevalence of dementia in PD to be 24.5% [13]. Subsequent studies have reported similar figures of 20–30% [14–16]. Despite the wide variation in the methodology used, the consistency of the rate found suggests it is close to the true proportion of dementia in PD. The systematic review found the prevalence of PDD as a percentage of all dementia cases to be 3.6% [13]. The lifetime prevalence of dementia in PD has also been studied, with 83% of PD patients surviving 20 years developing dementia [17], suggesting that dementia will eventually affect the vast majority of PD patients.

Neuropathological studies report that DLB comprises up to 15–20% of cases of dementia [17, 18], although such cohorts are invariably subject to small sample sizes and selection bias [19, 20]. Furthermore, concomitant Alzheimer’s disease (AD) and DLB pathology of varying severity has been found in post-mortem dementia cases, with no clear correlation as yet found with clinical phenotypes of AD or DLB [21]. In addition, many studies fail to correlate clinical data with pathological findings, describing DLB or PDD cases together under the category of LBD. Nevertheless, the 15–20% described in such studies is higher than the reported combined prevalence of DLB (4.2%) and PDD (3.6%) found clinically.

The clinical prevalence of DLB and PDD therefore remains unclear. We aimed to investigate the prevalence in a case series of DLB and dementia in PD across two distinct geographical sites. By employing an identical methodology in two comparable populations, we aimed to identify the rate of diagnosis of these dementias by clinicians in routine practice and better understand the variation in reported LBD diagnosis rates.

## Methods

We investigated prevalence in a case series to determine the clinical prevalence of DLB and PDD.

For assessing DLB, nine participating psychiatry of old age/memory clinic services in the UK were identified across four NHS hospital trusts, spread across two distinct geographical areas: East Anglia (EA, *n* = 2 trusts) and North-East England (NE, *n* = 2 trusts). Services were chosen by the research team in order to compile a cohort generalisable to that seen in routine clinical practice and included those serving both urban populations and mixed urban and rural populations. Among these were multidisciplinary teams serving urban areas (*n* = 2), serving rural areas (*n* = 1), and serving a mixture of both urban and rural populations (*n* = 6). One service was a tertiary memory clinic combining psychiatry and neurology expertise, and another incorporated a tertiary DLB clinic within a larger secondary care resource. All other services (*n* = 7) were secondary care organisations. Two clinics were closely affiliated with large teaching hospitals, the remaining seven with smaller district hospitals or community teams. For PDD, five PD or movement disorder clinics, each from a separate NHS trust (EA, *n* = 3 trusts; NE, *n* = 2 trusts) were sampled. These consisted of two geriatric medicine services and three which combined geriatric medicine and neurology expertise, serving urban (*n* = 2) and mixed urban and rural (*n* = 3) populations. None of these services incorporated specialist tertiary clinics.

The research team reviewed the notes of all subjects seen in services to identify patients with a diagnosis of dementia (for DLB prevalence), and those with a diagnosis of PD (for PDD prevalence), over a fixed 18-month period within a 2-year window from January 2013 to December 2014. Clinical diagnosis, as documented by the practitioner reviewing each patient within respective services, was recorded for each subject, as were age, gender, cognitive score, and date of diagnosis. For the DLB/dementia part of the study, dementia subtype, as determined by the clinician, was recorded. For the PDD/PD part of the study, the dates of diagnosis of both PDD (where applicable) and PD were recorded. Cases were coded as incident (dementia first diagnosed within the 18-month study period) or prevalent (dementia diagnosed prior to the study period, but the subject attended the service during the 18-month window). Patients who attended more than one participating service were included only in the service in which they were first seen. Permission was granted by the UK Confidentiality Advisory Group to collect these limited data from the clinical notes of all patients attending these services without the requirement of informed consent. Ethical approval for the study was also awarded by an NHS Regional Ethics Committee.

Statistical analysis was performed using SPSS 24.0 for Windows. Confidence intervals for prevalence in a case series were calculated using the Wilson method. Mean values and proportions were analysed using Student’s *t* test for independent samples and the χ^2^ test respectively. The Mantel–Haenszel χ^2^ test was used to test for a relationship between stratified age group and DLB prevalence. Non-parametric Spearman’s rank correlation was used to test for the correlation between the age at PD and the time to the onset of dementia, as the latter showed a non-normal positively skewed distribution. For each test statistic, *p* < 0.05 was regarded as statistically significant.

DLB prevalence in this case series was calculated as the percentage of DLB cases amongst the total number of dementia cases identified. PDD prevalence in the case series was calculated as the number of PD cases diagnosed with dementia, divided by the entire PD population seen during the screening period.

We approached a subset of patients with DLB and PDD, as well as cases matched by age (< 3 years) and gender to patients with non-DLB and PD diagnoses respectively, for consent to access their clinical notes in greater detail. DLB and non-DLB dementia cases were also matched by MMSE score (< 5 points). A panel of three expert clinicians reviewed clinical documentation and applied consensus criteria to each case. This method represents the accepted gold standard to *post-mortem* diagnosis, and has been validated against autopsy and imaging measures [22].

## Results

### DLB in psychiatry of old age services

**Fig. 1:**
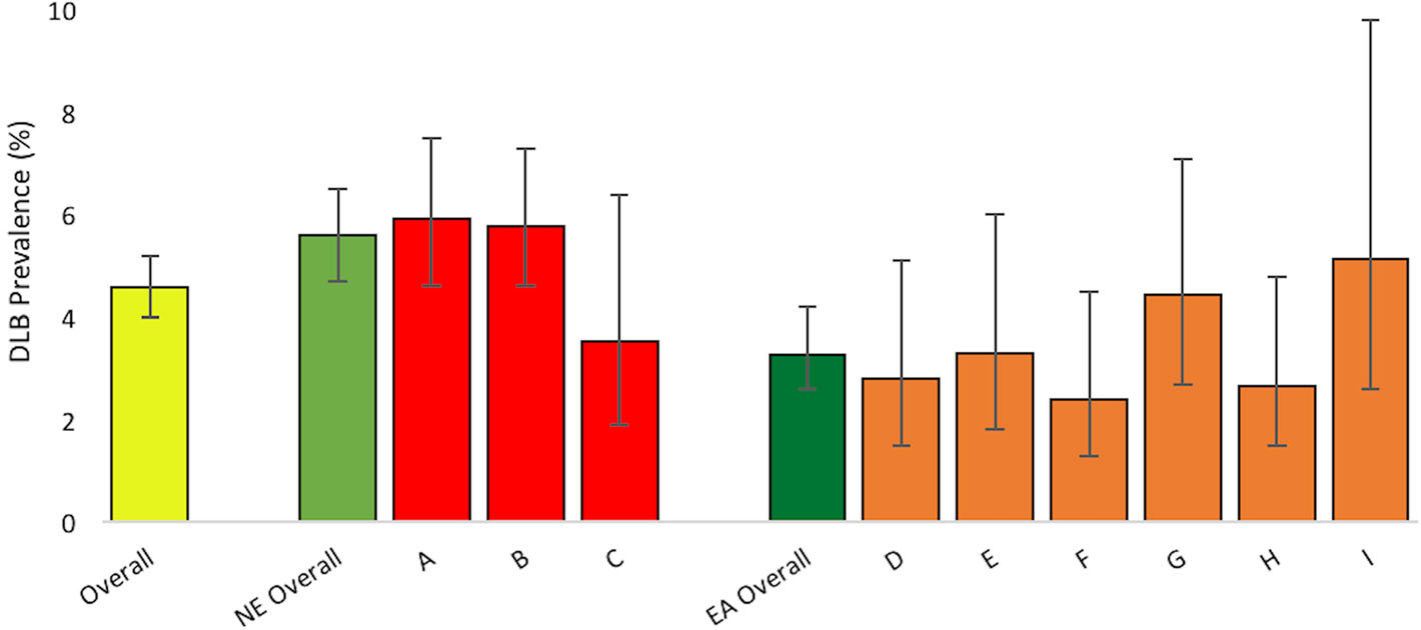
DLB prevalence by region and service. DLB dementia with Lewy bodies, EA East Anglia, NE North-East England, A–I services

The research team reviewed the case notes of 9449 individual patients, of whom 4504 (47.6%) had a dementia diagnosis (Fig. 1, Table 1), other diagnoses being mainly functional psychiatric disorders (such as depression) or cognitive problems falling short of dementia (such as mild cognitive impairment). Patients with DLB comprised 4.6% (95% CI 4.0–5.2%) of all dementia cases. Prevalence in individual services ranged from 2.4 to 5.9%, and was significantly higher among NE services (5.6%; 95% CI 4.8–6.5%; 70% greater) than in EA services (3.3%; 95% CI 2.6–4.2%; χ^2^ = 13.6, *p* < 0.01). No significant variation in prevalence was observed within each region (NE, χ^2^ = 2.54, *p* = 0.28; EA, χ^2^ = 4.88, *p* = 0.28).

**Table 1:** DLB prevalence and incidence by region and service

Service	Dementia (all subtypes)		DLB			
	Prevalent	Incident	Prevalent	% of prevalent dementia cases (95% CI)	Incident	% of incident dementia cases (95% CI)
A	1115	548	66	5.9 (4.7–7.5)	35	6.4 (4.6–8.8)
B	1178	637	68	5.8 (4.6–7.3)	36	5.7 (4.1–7.7)
C	282	106	10	3.5 (1.9–6.4)	4	3.8 (1.5–9.3)
North-East England	2575	1291	144	5.6 (4.8–6.5)	75	5.8 (4.7–7.2)
D	355	204	10	2.8 (1.5–5.1)	9	4.4 (2.3–8.2)
E	302	169	10	3.3 (1.8–6.0)	7	4.1 (2.0–8.3)
F	377	186	9	2.4 (1.3–4.5)	5	2.7 (1.2–6.1)
G	361	212	16	4.4 (2.7–7.1)	10	4.7 (2.6–8.5)
H	378	357	10	2.7 (1.4–4.8)	10	2.8 (1.5–5.1)
I	156	150	8	5.1 (2.6–9.8)	7	4.7 (2.3–9.3)
East Anglia	1929	1278	63	3.3 (2.6–4.2)	48	3.8 (2.8–4.9)
Overall	4504	2569	207	4.6 (4.0–5.2)	123	4.8 (4.0–5.7)

*CI* confidence interval, *DLB* dementia with Lewy bodies

Incident DLB cases made up 4.8% (95% CI 4.0–5.7) of dementia cases diagnosed within our study window, ranging from 2.7 to 6.4%. Incidence was also higher in NE services than in EA services (5.8 vs 3.8; χ^2^ = 5.9, *p* < 0.02; 53% greater).

**Fig. 2:**
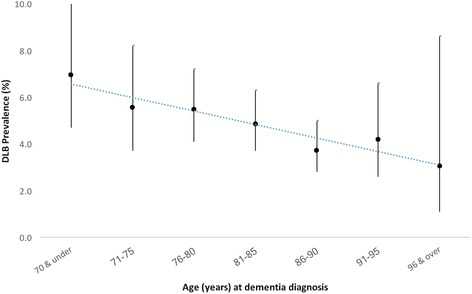
DLB prevalence and age at dementia diagnosis. DLB dementia with Lewy bodies

DLB prevalence was higher in men (χ^2^ = 24.8, *p* < 0.01) (Table 2). In addition, patients with DLB were significantly younger than their non-DLB counterparts (81.2 vs 82.4; *t*(4 502) = −2.1, *p* = 0.04), although the mean difference was just over a year, and this age difference was not seen in newly diagnosed cases. DLB prevalence in the case series also negatively correlated with stratified age (Mantel–Haenszel χ^2^ = 8.2, *p* < 0.01) (Fig. 2)*,* with similar findings for incident cases (Table 2) indicating that DLB was less commonly diagnosed in older people.

**Table 2:** Age and gender of DLB and non-DLB patients

	DLB	Non-DLB	*p*
Age at screening (± SD)			
Prevalent	81.3 (± 7.8)	82.4 (± 7.8)	0.04
Incident	81.8 (± 7.6)	82.1 (± 8.1)	0.59
Gender, male/female (% male)			
Prevalent	113/94 (54.6%)	1607/2690 (37.4%)	< 0.01
Incident	62/61 (50.4%)	958/1488 (39.2%)	0.01

*DLB* dementia with Lewy bodies, *SD* standard deviation

Seventy-five (75/207; 36.2%) DLB cases within the case series consented to a more detailed review of clinical documentation. The diagnosis made in clinical services concurred with that reported by expert clinician panel in 99% of cases (74/75). Expert panel also agreed with clinical diagnosis in 97% (72/74) of cases with non-DLB dementia.

### PDD in geriatric medicine and neurology services

The case notes of 2263 individual patients were examined, of whom 1563 (69.1%) had an idiopathic Parkinson’s disease diagnosis. PDD comprised 9.7% (*n* = 151, 95% CI 8.3–11.1%) of these PD cases. No significant variation was observed between regions: 8.3% in EA and 10.5% in NE (χ^2^ = 1.95, *p* = 0.16). There was also no significant variation found between all services, with PDD prevalence ranging from 4.5 to 11.0% (χ^2^ = 5.99, *p* = 0.20).

There was a male predominance in PD cases but no significant differences in gender found when comparing the two regions, in those with PDD, or when considering the larger cohorts of all PD patients (including PDD) between the regions (Table 3).

**Table 3:** Group demographics and differences between regions

Demographics	North-East England	East Anglia	Group difference
Gender (PDD), males/females	78/23	35/14	χ^2^ = 6.0, *p* = 0.44
Gender (all PD), males/females	587/385	328/260	χ^2^ = 3.2, *p* = 0.07
Age (years) at PDD onset, mean (± SD)	75.6 (± 6.7)	78.3 (± 7.3)	*t* = −2.1, *p* = 0.03
Age (years) at PD onset, mean (± SD)	70.3 (± 9.7)	73.1 (± 8.6)	*t* = 5.8, *p* < 0.01
Age at midpoint of screening period (all PD), mean (± SD)	76.9 (± 7.2)	78.7 (± 6.9)	*t* = 4.7, *p* < 0.01

*PD* Parkinson’s disease, *PDD* Parkinson’s disease dementia, *SD* standard deviation

However, both PD and PDD subjects were older in EA than in NE (PD mean difference of 2.8 years, *p* < 0.001; PDD mean difference of 2.7 years, *p* = 0.03).

**Fig. 3:**
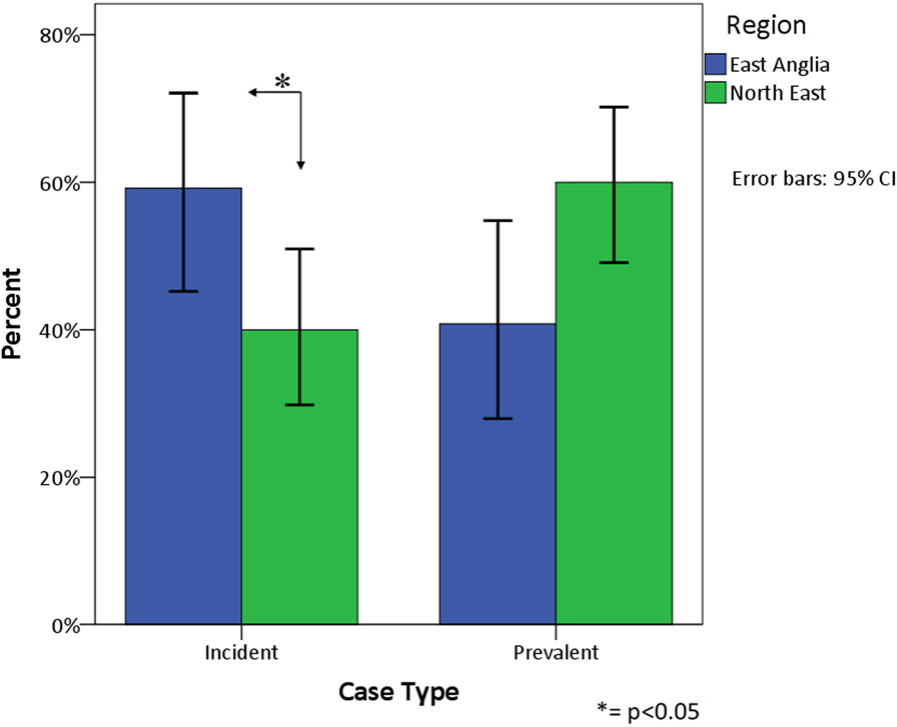
Percentage of cases of PDD diagnosed within our screening period compared to cases diagnosed before our screening period. Confidence interval (CI) calculated using standard (approximate method)

**Fig. 4:**
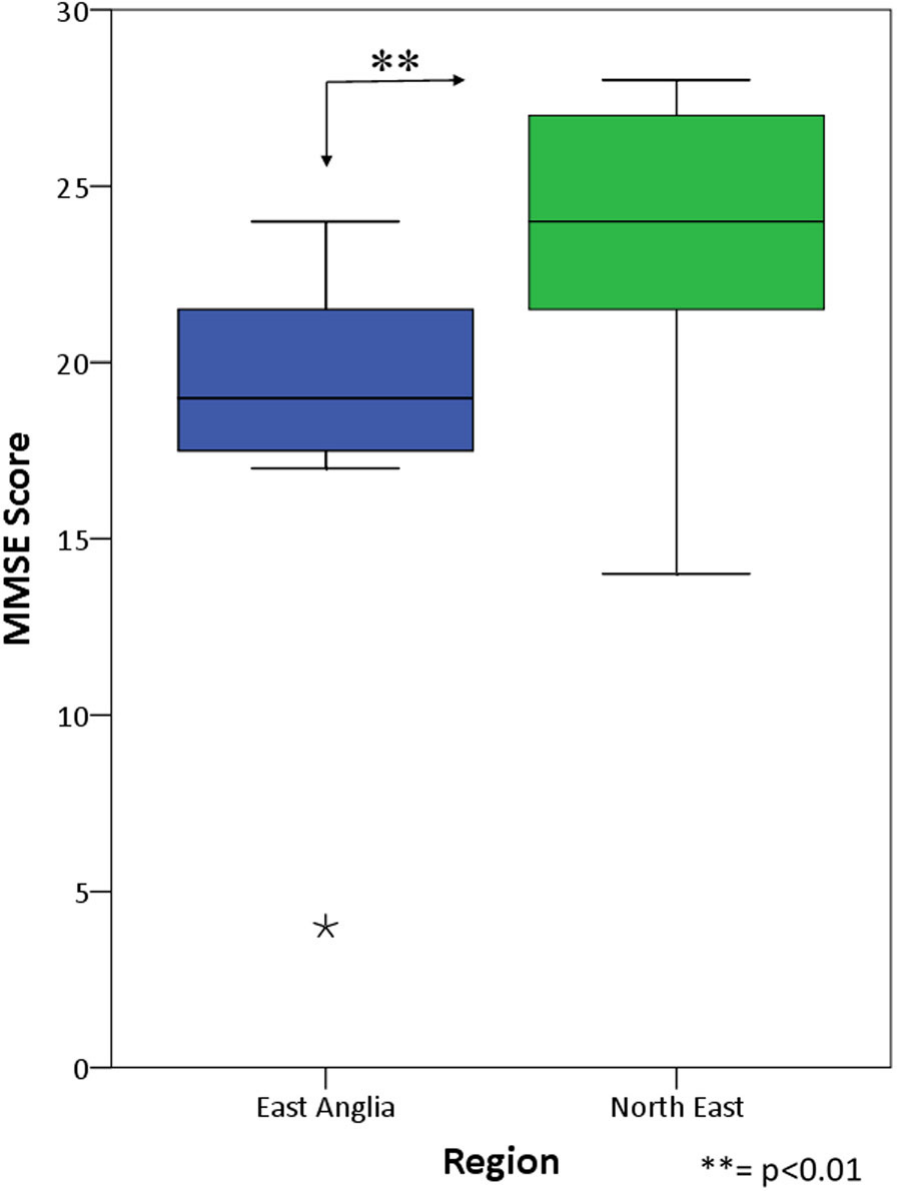
Comparison of cognitive scores at the time of PDD diagnosis between regions. MMSE Mini-Mental State Examination

Significantly more incident cases of PDD (newly diagnosed within our screening period) were found within EA compared to NE, comprising 59.1% of all PD cases in EA compared to 40.0% of cases in NE (χ^2^ = 4.49, *p* = 0.034; Fig. 3). In addition, significantly lower Mini-Mental State Examination (MMSE) scores at the time of PDD diagnosis were recorded in EA than in NE (Mann–Whitney *U*, *p* = 0.008; Fig. 4).

**Fig. 5:**
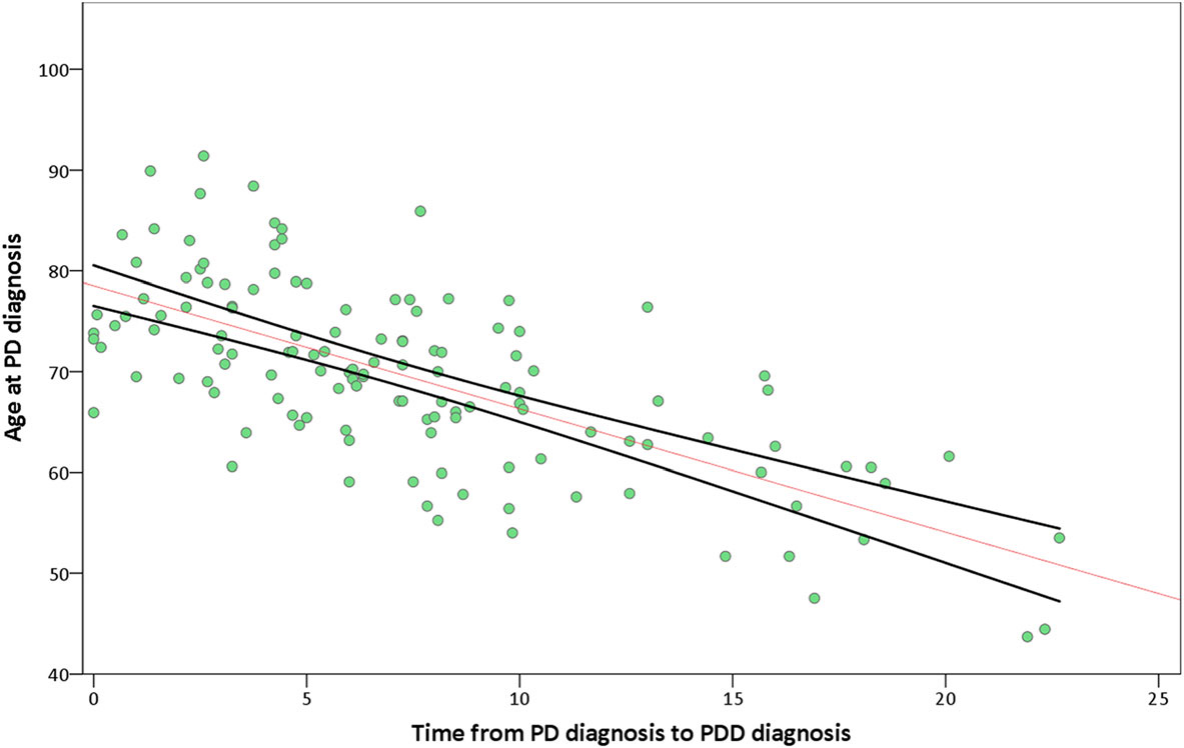
Negative correlation between age at initial diagnosis of PD and time before dementia onset. PD Parkinson’s disease, PDD Parkinson’s disease dementia

A highly significant inverse correlation between age at initial PD diagnosis and time until dementia onset (Spearman’s correlation, ρ = −0.66, *p* < 0.001) was also found in the PDD group as a whole (Fig. 5).

The diagnosis of the expert panel concurred with the diagnosis documented in the clinical notes in 97% of PDD cases consented for detailed notes review (37/38) and in 100% of recruited PD cases (35/35).

## Discussion

We found that DLB comprised 4.2% of all dementia cases in a representative clinical population in NHS secondary care services. This is a considerably lower figure than that cited by both neuropathological studies and previous meta-analyses [10, 18]. We also found dementia diagnosed in only 9.7% of cases of PD, much lower than the 20–30% seen in the systematic review [13] and subsequent population and clinic-based studies of PDD prevalence [14–16].

Our study was deliberately designed to determine the frequency of diagnoses in routine clinical services, and reflects current real-life practice for patients being assessed in specialist services within secondary care. Services were selected by the research team primarily on the basis of their generalisability to psychiatry of old age and neurology/geriatric medicine services, throughout the UK.

The most likely reason that rates found in our cohorts are lower than those reported in meta-analysis of other hospital-referred populations, and indeed nearer to community-based estimates, is probably to be found in the methodology employed. Our study was based upon scrutiny of routine clinical records from services receiving mainly community-based referrals. This cohort therefore represents a broader, more generalisable dementia population than those investigated in prevalence studies conducted within specialist centres that often show larger prevalence rates.

Nevertheless, our observed range in prevalence in a case series likely also reflects a lower rate of disease detection, rather than true disease prevalence in some populations. This is supported by the differences in prevalence of DLB observed between our NE and EA cohorts, and the wide range in rates observed in neighbouring services within the same region. This variation in detection may be related to a number of factors; the effect on medical education, training, and service development of Newcastle University’s long history of LBD research may have contributed to higher rates in NE. Varying sensitivity to core DLB features may play a role in detection; Walker *et al.* [23] noted that prevalence studies incorporating a neurological examination reported higher prevalence rates of DLB. It is also possible that not all practitioners comprising participating services are fully aware of consensus criteria, but the high level of agreement between diagnoses made within services and those made by the expert panel (98%) would suggest that consensus diagnostic guidelines are in routine use in participating services.

Despite our belief that our findings represent variation in DLB detection, variation in true disease prevalence cannot be entirely ruled out. Environmental factors or a combination of environmental factors in the pathogenesis of DLB have been proposed [24]. It is not possible to discount the possibility that the variation in regional diagnostic rates seen within this study simply reflect the degree of exposure to causative or precipitating biological factors, but the intra-regional variation which was also seen would argue against this.

Contrary to the findings of the meta-analysis, which reported a positive relationship between age and DLB prevalence (although this was not statistically significant), we identified an inverse correlation between these two factors, and found the mean age of DLB patients at diagnosis to be lower than that of non-DLB dementia patients. This may be a reflection of a more aggressive course and increased mortality in DLB, or that DLB becomes less common clinically with advancing age as other pathologies become more prevalent leading to a mixed pathological and clinical picture. Our study design and information systems did not allow us access to accurate mortality data, although increased mortality in DLB has been described [6].

DLB was also more prevalent among men than women in our cohort, a finding which also conflicts with the lack of significant association identified in meta-analysis [10]. A male preponderance has been observed in neuropathological DLB samples [25] but population samples have both supported and refuted this hypothesis [26, 27]. Our very large sample size and multi-servicing sampling make our data the strongest support for a male preponderance of DLB from clinical samples to date.

Dementia prevalence in our PD cohorts was much lower than has been reported previously. A variation in prevalence of dementia was not identified between regions, yet higher age and lower MMSE scores at diagnosis of dementia suggest that dementia is diagnosed later on in the disease in EA. However, as the age at PD diagnosis was also older in EA, once again the possibility that there may be an environmental factor driving earlier onset in NE cannot be discounted. Another reason behind the difference in age may be the differences in life expectancy between the regions – the latest figures show this to be 80.4/83.8 years (male/female) in EA and 78.0/81.7 years in NE [28] – similar to the age differences we observed between the two regions in the study. It is, however, possible that clinicians in the NE region have a lower threshold for making both diagnoses. It should also be noted that the mean age at the mid-point of our screening period across both regions was 77.6 years and was similar to the median of the mean ages in studies analysed in the systematic review by Aarsland *et al.* (74.9 years) [13].

The strong inverse correlation between age at onset of PD and the time to diagnosis of dementia is consistent with age being a risk factor for PDD [29].

As with DLB, the most likely cause of the lower prevalence rate of PDD in our case series is because we have reported the observed rate of diagnosis of PDD as made by clinicians in routine practice. Previous studies have sought to identify dementia specifically in their PD populations using standardised diagnostic tools. Although clinical diagnoses agreed with those made by our independent clinician panel in 99% of PDD and PD cases, it is likely that our findings reflect lower detection rates of PDD within the PD population.

A lower rate of diagnosis in clinical practice has important implications for the patients and their carers who benefit from a diagnosis being made. The development of dementia has a profound effect on the patient and carer, and allows for the provision of support services to cater for these. Dementia leads to loss of insight, poor judgement, poor financial decision-making, increased carer stress, impaired driving skills, and an increased falls risk, amongst other difficulties [17]. Healthcare providers would also need to adapt their services to cater for a higher population of their patients experiencing the difficulties of having dementia.

Strengths of this study include the very large sample size compared to previous studies, its multi-site nature (when previous estimates have usually involved only single sites), its representativeness, in that access to all cases within a service was allowed, and, since we used clinically made diagnoses, its clinical relevance. Potential limitations include the fact that we could not compare diagnostic rates made by clinicians with “true” prevalence, which would have required full clinical examination of all 12,500 cases and would not have been possible. Another important limitation of the study is that our methodology permitted investigation of DLB and PDD prevalence as determined by primary clinical dementia syndrome alone. We were therefore unable to determine the contribution of co-existing AD neuropathology in such cases, although no mechanism currently exists to accurately determine such cases on the basis of clinical presentation [21].

## Conclusion

Our study identified clinical prevalence rates of DLB and PDD in a case series considerably lower than that reported by clinical epidemiological cohorts and neuropathological studies. Importantly, we observed significant differences in the rates of DLB diagnosis among different regions, and a preponderance of DLB among males and younger patients. We found no such regional variations in prevalence amongst our clinical PDD population, but did find that PDD cases in EA were older, with a lower MMSE score, at the point of dementia diagnosis. Although our observation of regional variation in diagnosis could be attributed to different patterns of disease prevalence, a more likely explanation is that varying clinical diagnostic practices produce differences in DLB and PDD detection, rather than true disease prevalence.

Since it is important to accurately recognise and diagnose both DLB and PDD to optimise clinical care and management, and service delivery, and to allow more accurate prognosis, methods by which diagnostic rates might be improved should be tested. This might include the introduction of standardised assessments and scales to facilitate accurate recognition of DLB and PDD, including widespread use of the new DLB criteria [3], instruments such as the Lewy body composite risk score [30], or the DLB/PDD diagnostic toolkits [31].

## Abbreviations

AD: Alzheimer’s disease
DLB: Dementia with Lewy bodies
EA: East Anglia
LBD: Lewy body dementia
MMSE: Mini-Mental State Examination
NE: North-East England
NHS: National Health Service (UK)
PD: Parkinson’s disease
PDD: Parkinson’s disease dementia
UK: United Kingdom
